# IL-18 binding protein (IL-18BP) as a novel radiation countermeasure after radiation exposure in mice

**DOI:** 10.1038/s41598-020-75675-5

**Published:** 2020-10-29

**Authors:** Xianghong Li, Wanchang Cui, Lisa Hull, Li Wang, Tianzheng Yu, Mang Xiao

**Affiliations:** 1 Radiation Countermeasures Program, Armed Forces Radiobiology Research Institute, Uniformed Services University of the Health Sciences, 4555 South Palmer Road, Bethesda, MD 20889-5648 USA; 2 Consortium for Health and Military Performance, Department of Military and Emergency Medicine, Uniformed Services University of the Health Sciences, Bethesda, MD USA

**Keywords:** Biologics, Biological techniques, Cell biology, Drug discovery

## Abstract

Recent studies suggested that radiation exposure causes local and systemic inflammatory responses and induces cell and tissue damage. We have reported that IL-18 plays an important role in radiation-induced injury. Here, we demonstrate that IL-18 binding protein (IL-18BP), a natural antagonist of IL-18, was significantly increased (1.7–63 fold) in mouse serum on day 1 after 0.5–10 Gy TBI. However, this high level of IL-18BP was not sufficient to neutralize the active IL-18 in irradiated mice, resulting in a radiation dose-dependent free IL-18 increase in these mice’s serum which led to pathological alterations to the irradiated cells and tissues and finally caused animal death. Administration of recombinant human (rh) IL-18BP (1.5 mg/kg) with single (24, 48 or 72 h post-TBI) or double doses (48 h and 5 days post-TBI) subcutaneous (SC) injection increased 30-day survival of CD2F1 mice after 9 Gy TBI 12.5–25% compared with the vehicle control treated group, respectively. Furthermore, the mitigative effects of rhIL-18BP included balancing the ratio of IL-18/IL-18BP and decreasing the free IL-18 levels in irradiated mouse serum and significantly increasing blood cell counts, BM hematopoietic cellularity and stem and progenitor cell clonogenicity in mouse BM. Furthermore, IL-18BP treatment inhibited the IL-18 downstream target interferon (IFN)-γ expression in mouse BM, decreased reactive oxygen species (ROS) level in the irradiated mouse heart tissues, attenuated the stress responsive factor GDF-15 (growth differentiation factor-15) and increased the intestine protector citrulline level in total body irradiated mouse serum, implicating that IL-18BP may protect multiple organs from radiation-induced inflammation and oxidative stress. Our data suggest that IL-18 plays a key role in radiation-induced cell and tissue damage and dysfunction; and for the first time demonstrated that IL-18BP counters IL-18 activation and therefore may mitigate/treat radiation-induced multiple organ injuries and increase animal survival with a wider therapeutic window from 24 h and beyond after lethal doses of radiation exposure.

## Introduction

The mechanisms of radiation-mediated multi-organ injury are not well understood and a few medical countermeasures are FDA approved to treat acute radiation injuries. Radiation causes cellular DNA damage leading to “danger signals” and antigen release. These damage-associated molecular patterns (DAMPs) are endogenous molecules released from damaged cells and are important proinflammatory causal factors which can result in immune cell activation, inflammation and death^1,2^. In addition, a massive release of radiation-induced proinflammatory cytokines induces cell apoptosis, senescence, pyroptosis or necrosis^3,4^. IL-1 family members IL-1β, IL-18, and IL-33 play key roles in inflammatory and immune responses and have been described as having significant influence on the pathogenesis of diseases^5^. We recently reported that IL-1β, IL-18, and IL-33 were upregulated in mouse thymus, spleen, and bone marrow (BM) after total-body irradiation (TBI)^6^. Of these cytokines, IL-18 was characterized by a significant and persistent increase in mouse, minipig, and nonhuman primate (NHP) serum and/or urine in a radiation dose-dependent manner and the highest levels of IL-18 were observed on day 3 after TBI, suggesting IL-18 plays important roles in radiation-induced injury^6,7^. IL-18 is a highly pleiotropic pro-inflammatory cytokine produced by various hematopoietic and nonhematopoietic cells, including endothelia cells (ECs), dendritic cells and macrophages and can propagate inflammation by promoting immune cell infiltration, leukocyte and lymphocyte activation and angiogenesis, and facilitates the transition from the innate to the adaptive immune response^8,9^.

IL-18 binding protein (IL-18BP) is a natural antagonist of IL-18^10,11^ which belongs to the immunoglobulin-like class of receptors and is not cleaved on the cell surface. IL-18BP has 4 isoforms (a, b, c, and d) in humans and 2 isoforms (c and d) in mice, the human isoforms a and c and mouse isoforms c and d can bind to the receptor-binding site of IL-18 to inhibit IL-18 at equimolar ratio^12,13^. IL-18BP binds to IL-18 with high affinity to block the IL-18 and IL-18R complex formation and subsequently inhibits IL-18 activation^9^. Under normal conditions, there is enough naturally occurring IL-18BP to keep a low level of free IL-18. However, in patients with certain inflammatory diseases and high level of IL-18 could cause the IL-18/IL-18BP balance disruption, leading to an increase in free and active IL-18, which in turn results in pathological inflammation. This imbalance of IL-18/IL-18BP can be associated with increased disease severity. More recently, Belkaya et al.^14^ reported a child who was homozygous for a private 40-nucleotide deletion in *IL-18BP* gene and could not produce IL-18BP and died upon infection with hepatitis A virus (HAV). The author suggested that the absence of IL-18BP caused imbalance of IL-18/IL-18BP and excessive NK cell activation by high level IL-18 resulted in uncontrolled killing of human hepatocytes.

The effects of IL-18 have been implicated in many diseases including autoimmune diseases^15^, cardiovascular diseases^16–18^, neurovascular EC damage^19^, infrarenal aortic occlusion-mediated renal injury^20^, hemophagocytic lyphohistiocytosis^21^, and inflammatory bowel disease^22^ in clinical and animal studies. IL-18BP and anti-IL-18 antibody have been used to neutralize IL-18 and treat these diseases. Novick et al.^23^ summarized 33 disease models in which inhibition of IL-18 activity by administration of either neutralizing anti-IL-18 antibodies or IL-18BP resulted in a reduction of disease severity. Recently, recombinant human IL-18BP or called Tadekinig Alfa has been used for a phase II clinical trial in Europe to treat adult onset Still’s disease patients^24^ and a phase III clinical trial with the experimental drug IL-18BP in patients carrying a mutation of the NOD-like receptor C4 (NLRC4) gene characterized by severe, life threatening systemic inflammation associated with extremely high levels of IL-18 (https://www.ab2bio.com). The increase of IL-18 in radiation injury has been also reported by many groups including us^6,7^. Shan et al.^25^ reported that TBI between 0.05 and 4 Gy resulted in radiation dose-dependent IL-12 and IL-18 secretion and increased TLR4-MD2 and MyD88 expression in mouse peritoneal macrophages. Furthermore, radiation-induced NLRP3 inflammasome activation and caspase-1 cleavage, correlated with apoptosis in mouse immune cells, have been reported^26^. Clinical radiocontrast administration for abdominal computed tomography (CT) caused increases of spot urine IL-18 levels^27^. The authors suggested that the increased IL-18 in patients’ urine can be used as an early parameter for kidney injury after radiocontrast administration. Thus, in the current study, we tested our hypotheses that the IL-18 inflammatory signal pathway plays a key role in radiation-induced cell and tissue damage and dysfunction. IL-18BP counters IL-18 activation and therefore may mitigate/treat IL-18-mediated multi-organ injury and increase survival after lethal doses of radiation.

## Materials and methods

### Ethics statement

Animals were housed in an Association for Assessment and Accreditation of Laboratory Animal Care (AAALAC)-approved facility at the Uniformed Services University of the Health Sciences (USUHS). All animal study procedures including housing, drug administration, irradiation, survival study and blood and tissue collection were reviewed and approved by the USUHS Institutional Animal Care and Use Committee (IACUC) and all experiments were performed in accordance with the USUHS-IACUC and the USUHS Department of Laboratory Animal Resources (DLAR) relevant guidelines and regulations.

### Mice and animal care

Twelve- to 14-week-old CD2F1 male mice (ENVIGO, Indianapolis, IN) were used according to the methods as previously described^28^. Mice were chosen randomly for each experimental group and housed in an AAALAC-approved facility at the USUHS. The animal study protocol was approved by the Institutional IACUC. Animal rooms were maintained at 20–26 °C with 30–70% humidity on a 12 h light/dark cycle. Commercial rodent chow (Harlan Teklad Rodent Diet 8604) was available ad libitum as was acidified water (pH = 2.5–3.0) to control opportunistic infections.

### Irradiation and drug

Mice received total-body irradiation (TBI) in a bilateral radiation field at AFRRI’s ^60^Co facility. The alanine/electron spin resonance (ESR) dosimetry system (American Society for Testing and Materials, Standard E 1607) was used to measure dose rates (to water) in the cores of acrylic mouse phantoms. Control animals were sham-irradiated, treated in the same manner as the irradiated animals except the ^60^Co source was not raised from the shielding water pool as previously described^29^. The midline tissue dose to the mice was 0.5, 1, 3, 5, 7, 8, 9, 9.4 or 10 Gy at a dose rate of 0.6 Gy/min. The day of irradiation was considered day 0.

Recombinant human IL-18BPa (rhIL-18BP, Cat No. 119-BP; R&D Systems, Minneapolis) was used in this study because human and murine IL-18BP isoforms are active across species^30^ and has been used in rat and mouse studies^20,21,31,32^. rhIL-18BP is a water soluble small molecule and was freshly prepared with saline. Saline was used as vehicle control in the animal studies. Drug-treated mice weighing 25 ± 2 g received 0.1 mL of rhIL-18BP, with injection volume adjusted for mice weighing more than 28 g. Control mice received 0.1 mL of vehicle. A single or two-dose subcutaneous (SC) injection of rhIL-18BP or vehicle was done aseptically at the nape of the mouse neck with a 23G needle at 24, 48 or 72 h (single dose) or 48 h + 5 days (two-dose) after radiation.

### rhIL-18BP toxicity study

This study followed the AFRRI drug screening program protocol^33^. The safe dose of rhIL-18BP for animal studies was selected according to the AFRRI toxicity study protocol (modified “up and down” procedure, UDP). A single dose of rhIL-18BP (0.25, 0.5, 1.0, 1.5, 2.0, 3.0 or 5.0 mg/kg body weight)^21,32^ or saline vehicle in 0.1 mL total volume was injected SC in mice (N = 6 mice/group) and animals were monitored for 14 days for any physical abnormalities including body weight loss, injection site reactions, and changes in behavior or activity level. Blood cell counts, spleen, lung, liver and kidney size and morphology were also examined at the end of toxicity study.

### Thirty-day survival studies

rhIL-18BP 1.5 mg/kg with single or two-dose schedule were used for the survival studies. For single-dose treatment, mice received the vehicle-control or rhIL-18BP-treatment at 24, 48 or 72 h post-TBI; for the two-dose treatment, mice received vehicle-control or rhIL-18BP-treatment at 48 h and day-5 (day 2 + 5) post-TBI. Mice were observed for 30 days to determine the survivability after 9.0 Gy (LD70/30) total-body radiation exposure (N = 20 mice/group). After 30 days, the survived animal’s tissues and blood were collected as described below.

### Mouse peripheral blood cell counts and serum and tissue preparation

All methods were developed in our group as described previously^3^. Mice were humanely euthanized for whole blood, serum and tissue collection. Euthanasia was carried out in accordance with the recommendations and guidelines of the American Veterinary Medical Association. The mice were deeply anesthetized prior to collect whole blood through a cardiac blood draw and confirmatory cervical dislocation was performed while the animal was still anesthetized in accordance with the approved IACUC protocol. The blood was immediately divided into two tubes. The samples in EDTA tubes were used for peripheral blood cell counts by a clinical hematoanalyzer (Element HT5, Heska Co. Loveland, Colorado), and samples in BD Microtainer Gold tubes were left unmoved on racks. Following 30 min coagulation at room temperature, the sera were well separated from the gel by 10 min-centrifugation at 10,000×*g*, collected and stored at – 80 °C for later study. Once blood collection from individual mouse and the mouse euthanasia were completed, mouse tissues were collected.

### Clonogenicity assay

BM cells were collected from mouse femurs and humeri after TBI. After erythrocytes were lysed with Erythrocyte Lysis Buffer (Qiagen GmbH, Hilden), total BM myeloid cell viability from each mouse was measured using Trypan blue staining. Clonogenicity of mouse BM cells was quantified in standard semisolid cultures in triplicates using 1 mL of Methocult GF + system (including SCF, IL-3, IL-6 and erythropoietin) for mouse cells (Cat # 03444, StemCell Technologies) according to the manufacturer’s instructions, as described previously^29^. Briefly, mouse BM cells from individual animal were seeded at 5 × 10^4^ or 3 × 10^5^ cells/dish in 35-cm cell culture dishes (BD Biosciences). Plates were scored for total colony forming units (CFUs) and colonies of erythroid burst-forming units (BFU-E), granulocyte–macrophage (CFU-GM), and mixed-lineage (CFU-GEMM) after culturing for 8–10 days at 37 °C in 5% CO_2_.

### Histological studies of mouse BM

Mouse sterna were fixed in Z-Fix (formaldehyde, methanol, ionized zinc buffer, Anatech Ltd., Battle Creek, MI, USA) for at least 24 h. Samples were decalcified (Cal-EX for 3 h) and sectioned longitudinally. After hematoxylin–eosin (HE) staining, slides were examined under the microscope. The bright field images of HE stained slides were acquired by Zeiss Axioscan.Z1 and analyzed using Zeiss Zen 2.5 (blue edition) and NIH ImageJ analysis program. The adipocytes in BM were scored under the higher power.

### BM immunohistochemistry (IHC) staining

Mouse sterna were fixed in 10% formalin, decalcified, embedded in paraffin, and cut into 5 µm sections per slide. For IHC staining, antigen retrieval was performed in Citrate Buffer, pH 6.0 at 100 °C for 20 min, followed by gradual cooling to room temperature. BloxAll solution (Vector Laboratories, Inc., Burlingame, CA, USA) was used to quench the endogenous peroxidase activity. Specimens were then blocked with 2.5% normal serum (Vector Laboratories, Inc.) and then incubated at 4 °C overnight with primary antibodies (anti-IL-18 antibody, Abcam ab207323; anti-IFN-γ antibody, Abcam ab9657; anti-IL-15 antibody, Abcam ab34674; anti-TNF-α antibody, R&D systems AF447; anti-IL-12/IL-35 p35 Antibody, R &D systems MAB6688; and rabbit IgG isotype control antibody, Abcam ab37415). Specimens were washed and then subsequently incubated with respective ImmPRESS HRP IgG Polymer Detection Kits (Vector Laboratories, Inc.). ImmPACT DAB Kit (Vector Laboratories, Inc.) was used to develop the substrate to the desired stain intensity. The slides were counterstained with hematoxylin and images were scanned using a Zeiss Axio Scan.Z1 system.

The IHC positive staining was quantified using ImageJ Fiji software according to a documented protocol^34^, 3 mice/group were examined. Three to five random areas were chosen from each bone marrow sample slide. The DAB color was deconvoluted and its threshold value was adjusted for the optimal representation of positive staining. Using the same setting, the percentage of positive staining area in each image was quantified. The average of positive staining from each animal was used for statistical analysis.

### Determine reactive oxygen species (ROS) levels in mouse heart tissues

ROS level in mouse heart tissues was determined using dihydroethidium staining (DHE, Invitrogen, Waltham, MA) as described previously^35^. Briefly, mouse heart was harvested, snap frozen in a Tissue-Plus OCT compound (Fisher Scientific, Hampton, NH), and cut in 5 µm sections. Cryostat sections were mounted on Superfrost Plus slides and stained with 5 µM DHE in PBS at room temperature for 10 min. Fluorescence images were viewed and acquired with a Nikon Eclipse Ti epifluorescence microscope equipped with a digital camera (Melville, NY). The images were analyzed using ImageJ software (NIH) and DHE fluorescence intensity was quantified as the ROS levels.

### IL-18, IL-18BP, GDF15 and citrulline quantitation by enzyme-linked immune sorbent assay (ELISA) or colorimetric kits

ELISA kits for mouse IL-18 from Medical Biological Laboratories (MBL), mouse IL-18BP from Cloud-Clone Corp, human IL-18BP and mouse GDF15 from R&D Systems were purchased. Cytokine levels in serum were determined in duplicate following assay instructions provided by the manufacturers (6 mice/group). Briefly, mouse serum 30 µL, 10 µL, 10 µL or 0.5–5 µL from each sample were incubated for 2 h in microwells coated with anti-mouse IL-18, IL-18BP, GDF-15, or anti-human IL-18BP antibody, respectively. After washing, peroxidase conjugated second antibody specifically to the corresponding antigen was added to the microwells and incubated for 1 h. After another washing, the substrate reagent was mixed with the chromogen and incubated for 30 min. An acid solution was added to each microwell to terminate the enzyme reaction and to stabilize the color development. The optical density (OD) of each microwell was measured at 450 nm using a microplate reader. The minimum detection limits for mouse IL-18, mouse IL-18BP, human IL-18BP, and GDF-15 were 9.8 pg/mL, 45 pg/mL, 26.6 pg/mL, and 7.8 pg/mL, respectively. No cross-reaction between human and mouse IL-18BP ELISAs were observed.

Citrulline Colorimetric Kit was purchased from CELL BIOLABS, INC. The citrulline levels in the mouse serum were determined in duplicate following assay instruction provided by the manufacturer. Briefly, 5 µL of mouse serum from each sample was treated with SDS and Proteinase K to release free citrulline residues. Assay reagent was added which reacted with citrulline to produce a chromophore and the absorbance was read at 540 nm. The minimum detection limit for citrulline was 37.5 µM.

### Calculation of free IL-18

According to the published methods, free IL-18 was calculated by applying the law of mass action based on the following parameters: total IL-18 and total IL-18BP concentrations (each determined by the corresponding ELISA), a 1:1 stoichiometry in the complex of IL-18 and IL-18BP and complex dissociation constant (KD) of 0.4 nM^12,13,36^ and compared with a new calculation method which suggested the KD between 30 and 50 pM (40 pM)^37^.

### Statistical analysis

The 30-day survival of mice was analyzed using Kaplan–Meier analysis. Fisher's exact test was used to compare survival among groups at the end of 30 days after TBI, and p < 0.05 will be taken as statistically significant. For other cell biology data, differences between means were compared by analysis of variance (ANOVA) and Student’s *t* tests. p < 0.05 was considered statistically significant. Results were presented as means ± standard deviations.

## Results

### Radiation induced imbalance of IL-18 to IL-18BP and increased free-IL-18 in mouse serum

We have reported the radiation-induced IL-18 in mouse serum from 1–13 days after irradiation and reached a peak on day 3 after TBI, whereas IL-18BP was increased on day 1 and quickly dropped toward the baseline^38^. However, the severity of radiation injury may depend on whether the IL-18BP is sufficient to neutralize the active IL-18^36^. In this study, we calculated the free active-IL-18 (free IL-18) in mouse serum on day 1 and day 3 after 0.5–10 Gy TBI according to the published methods indicated in Materials and Methods from Novick et al., with KD = 400 pM^11–13^ and Girard et al., with KD = 40 pM (30–50 pM)^37^. Mouse serum samples from 0 Gy control, and 0.5, 1, 3, 5, 7, 8, 9 or 10 Gy TBI mice on day 1 and day 3 after irradiation were collected and levels of IL-18 and IL-18BP in serum of individual mice were measured in parallel by ELISA. Table 1 summarizes the average IL-18BP, total IL-18 and free IL-18 levels in mouse serum after 0 or 0.5–10 Gy of TBI and data were from two independent experiments (6 mice/group in each experiment × 2 experiments = 12 mice/group). The concentration of total IL-18 in mouse sera from the sham-irradiated control group (0 Gy) was 17.6 ± 1.75 pg/mL and IL-18BP was 2,797 ± 0.51 pg/mL, resulted in a 14.99 ± 1.13 pg/mL (Novick’s method) and 6.44 ± 1.1 pg/mL(Girard’s method) of free IL-18 in this group, respectively. Radiation at 0.5 and 1 Gy increased IL-18BP but not total IL-18 levels, and the elevated free IL-18 was not observed in mouse serum compared with sham-irradiated controls. Whereas, radiation > 3 Gy significantly increased the total IL-18 levels in mouse serum on day 1 and continually increased on day 3 after TBI in a radiation dose-dependent manner. Radiation-induced IL-18BP expression was observed on day 1 after 0.5–10 Gy irradiation; the endogenous IL-18BP was dramatically increased from 2.8 ng/mL of sham-irradiated controls up to 176.6 ng/mL in the 10 Gy TBI group, resulted in no increase of free IL-18 levels in irradiated samples on day 1 compared with sham-irradiated controls. However, 3 days after irradiation, IL-18BP levels had dropped sharply and free-IL-18 was significantly increased compared with the sham-control level by both calculation methods, which was correlated with radiation-induced mortality. Although the free IL-18 in serum calculated by Girard’s method was significantly lower than the Novick’s method, the calculated levels of free IL-18 difference between these two calculation methods did not change our conclusion and further confirmed that the free IL-18 levels in mouse serum were radiation dose-dependent.

**Table 1:** Average levels of free IL-18 in mouse serum after total-body irradiation.

		IL-18BP (pg/mL)	IL-18 (pg/mL)	Method-1: Kd = 400 pM			Method-2: Kd = 40 pM		
				free IL-18 (pg/mL)	Fold change	p value	free IL-18 (pg/mL)	Fold change	p value
0 Gy		2,797 ± 0.51	17.6 ± 1.75	14.98 ± 1.13			6.44 ± 1.10		
0.5 Gy	D1	4,814.21 ± 2.75	8.22 ± 3.21	6.32 ± 2.98	0.42		2.06 ± 2.01	0.28	
	D3	3,674.47 ± 3.63	11.51 ± 2.39	9.36 ± 3.01	0.62		3.50 ± 2.18	0.48	
1 Gy	D1	3,976.268 ± 8.89	12.58 ± 2.63	10.08 ± 3.76	0.67		3.62 ± 2.25	0.49	
	D3	4,033.15 ± 10.01	7.63 ± 4.09	6.10 ± 7.05	0.41		2.17 ± 3.05	0.29	
3 Gy	D1	34,448.78 ± 1.09	43.68 ± 10.42	13.87 ± 5.76	0.93		1.94 ± 1.06	0.26	
	D3	3,308.97 ± 3.78	89.65 ± 13.55	74.42 ± 8.67	4.96**	0.001	30.03 ± 4.77	4.07**	0.001
5 Gy	D1	72,041 ± 4.22	47 ± 7.16	8.55 ± 5.69	0.57		1.02 ± 0.68	0.14	
	D3	6,139 ± 2.06	95.5 ± 15.03	69.20 ± 8.55	4.62**	0.0065	20.18 ± 4.21	2.74*	0.02
7 Gy	D1	153,567.9 ± 26.75	56 ± 10.11	5.29 ± 18.43	0.35		0.58 ± 0.64	0.08	
	D3	11,334.8 ± 6.06	210 ± 16.02	123.79 ± 4.04	8.26**	< 0.0001	26.82 ± 3.67	3.64**	0.001
8 Gy	D1	130,839.1 ± 10.33	47.3 ± 5.19	5.16 ± 7.76	0.34		0.57 ± 0.99	0.08	
	D3	19,836 ± 5.76	421.3 ± 4.76	190.83 ± 5.26	12.73**	< 0.0001	32.77 ± 3.98	4.45**	0.001
9 Gy	D1	94,603.4 ± 16.22	83.4 ± 6.15	12.08 ± 11.19	0.81		1.39 ± 1.11	0.19	
	D3	35,237.4 ± 7.61	611.8 ± 4.22	194.57 ± 5.92	12.98**	< 0.0001	27.54 ± 3.64	3.74**	0.001
10 Gy	D1	176,555 ± 10.91	84.2 ± 6.14	7.00 ± 8.53	0.47		0.76 ± 0.89	0.1	
	D3	57,513 ± 7.68	820.1 ± 7.33	182.00 ± 7.51	12.14**	< 0.0001	22.88 ± 3.93	3.10**	0.01

Summarizes the average free IL-18 levels in mouse serum 0 or 0.5–10 Gy of TBI. Data were from two independent experiments (6 mice/experiment × 2 = 12 mice/group).

*p < 0.05; **p < 0.01; Gamma irradiated vs. sham (0 Gy) irradiated animal. Method-1: Based on Novick et al.^13^; Method-2: Based on Girard et al.^37^.

### IL-18BP administration increased survival of mice after total-body irradiation

Next, we tested our hypothesis that administration of rhIL-18BP in mice after TBI could boost the levels of IL-18BP in mouse serum and inhibit the active IL-18-induced inflammation and increase survival in irradiated mice. First, the toxicity study on rhIL-18BP was performed according to our institute’s protocol. A single dose of rhIL-18BP (0.25, 0.5, 1.0, 1.5, 2.0, 3.0 or 5.0 mg/kg of body weight) or saline as vehicle-control in 0.1 mL total volume was injected SC in mice (N = 6 mice/group) and animals were tracked 14 days. No changes of body weight (Table 2) and blood cell number including the total white blood cells (WBC), neutrophils (NEU), lymphocytes (LYM), mononuclear cells (MONO), red blood cells (RBC), hemoglobin (HGB) and platelets (PLT) (Table 3) were correlated with rhIL-18BP doses in comparison with vehicle-injected mice. Although some blood parameters in Table 3 were significantly different from the vehicle group, there was no dose-dependent effect of rhIL-18BP, and those values were still in the normal range, suggesting no toxicity of the drug. In addition, no morphology of organs and behavior changes between rhIL-18BP injected and vehicle injected mice were observed, and no infections or local reactions were noted at the sites of injection (data not shown).

**Table 2:** Body-weight (g) measurement in CD2F1 mice after IL-18BP or vehicle treatment.

Treatment	Pre-injection	D1	D2	D3	D4	D7	D9	D11	D14
Vehicle	28.7 ± 0.9	28.4 ± 1.0	28.3 ± 1.3	28.0 ± 1.0	28.3 ± 1.0	28.6 ± 0.7	28.8 ± 0.9	29.2 ± 1.0	28.8 ± 1.0
**IL-18BP**									
0.25 mg/kg	28.4 ± 3.8	28.1 ± 3.5	28.3 ± 3.7	28.4 ± 3.7	28.7 ± 3.8	29.1 ± 3.8	29.1 ± 4.0	29.9 ± 4.2	29.5 ± 4.2
0.5 mg/kg	27.1 ± 1.3	26.7 ± 1.5	26.4 ± 1.3	26.5 ± 1.3	27.4 ± 1.3	27.5 ± 1.5	27.3 ± 1.6	28.0 ± 1.7	28.9 ± 1.7
1.0 mg/kg	28.5 ± 1.8	28.4 ± 2.0	27.9 ± 2.0	27.9 ± 2.0	28.0 ± 2.0	28.2 ± 1.8	28.6 ± 1.9	28.6 ± 1.9	28.7 ± 1.8
1.5 mg/kg	28.4 ± 2.0	28.0 ± 2.1	28.0 ± 2.0	28.1 ± 1.7	28.5 ± 1.9	28.6 ± 1.6	28.7 ± 1.6	29.0 ± 1.5	29.0 ± 1.4
2.0 mg/kg	29.2 ± 1.6	29.2 ± 1.6	29.0 ± 1.6	29.0 ± 1.8	29.5 ± 1.8	29.6 ± 2.0	29.7 ± 2.1	30.1 ± 2.1	29.9 ± 2.2
3.0 mg/kg	29.1 ± 1.3	29.3 ± 1.5	28.9 ± 1.4	28.5 ± 1.4	29.3 ± 0.7	28.5 ± 1.4	28.9 ± 1.6	29.1 ± 1.5	29.2 ± 1.8
5.0 mg/kg	29.2 ± 1.6	29.0 ± 1.8	28.8 ± 1.7	28.5 ± 1.8	29.2 ± 1.2	28.5 ± 1.7	28.8 ± 1.7	29.2 ± 1.6	29.3 ± 1.8

Subcutaneous injection, no significant body weight changes before and after treatment with either IL-18BP or vehicle (N = 6).

**Table 3:** Blood cell counts in CD2F1 mice 14 days after IL-18BP or vehicle treatment.

Treatment	WBC (× 10^3^)	NEU (× 10^3^)	LYM (× 10^3^)	MONO (× 10^3^)	RBC (× 10^3^)	HGB (g/dL)	PLT (× 10^3^)
Vehicle	6.55 ± 2.37	1.54 ± 0.66	4.34 ± 1.55	0.16 ± 0.08	8.82 ± 1.70	12.78 ± 2.94	1001.00 ± 217.74
**IL-18BP**							
0.25 mg/kg	4.83 ± 0.71	1.04 ± 0.16	3.38 ± 0.60	0.09 ± 0.06	9.22 ± 0.48	13.07 ± 0.56	1055.67 ± 81.99
0.5 mg/kg	4.36 ± 1.73	0.98 ± 0.13	3.55 ± 1.67	0.05 ± 0.04	7.91 ± 2.50	10.20 ± 3.65 *	921.67 ± 382.38
1.0 mg/kg	6.51 ± 1.24	1.45 ± 0.43	4.62 ± 0.84	0.17 ± 0.09	8.93 ± 0.56	13.40 ± 0.95	960.17 ± 142.65
1.5 mg/kg	6.32 ± 1.63	1.02 ± 0.53	4.74 ± 1.07	0.14 ± 0.09	8.60 ± 0.59	13.07 ± 0.76	1075.33 ± 114.34
2.0 mg/kg	3.99 ± 1.09 *	0.64 ± 0.20 *	3.60 ± 0.86	0.11 ± 0.05	7.77 ± 2.18	11.85 ± 3.28	775.17 ± 267.59
3.0 mg/kg	5.55 ± 1.32	1.26 ± 0.41	3.89 ± 0.88	0.15 ± 0.02	9.35 ± 0.37	14.20 ± 0.3	1008.00 ± 76.28
5.0 mg/kg	7.99 ± 0.63	1.54 ± 0.30	5.88 ± 0.44	0.23 ± 0.12	9.59 ± 0.38	14.43 ± 0.55	1010.33 ± 179.73

Mouse blood were collected and blood cell counts were evaluated. IL-18BP-treated vs. vehicle control-treated. *p < 0.05: (N = 6).

**Figure 1:**
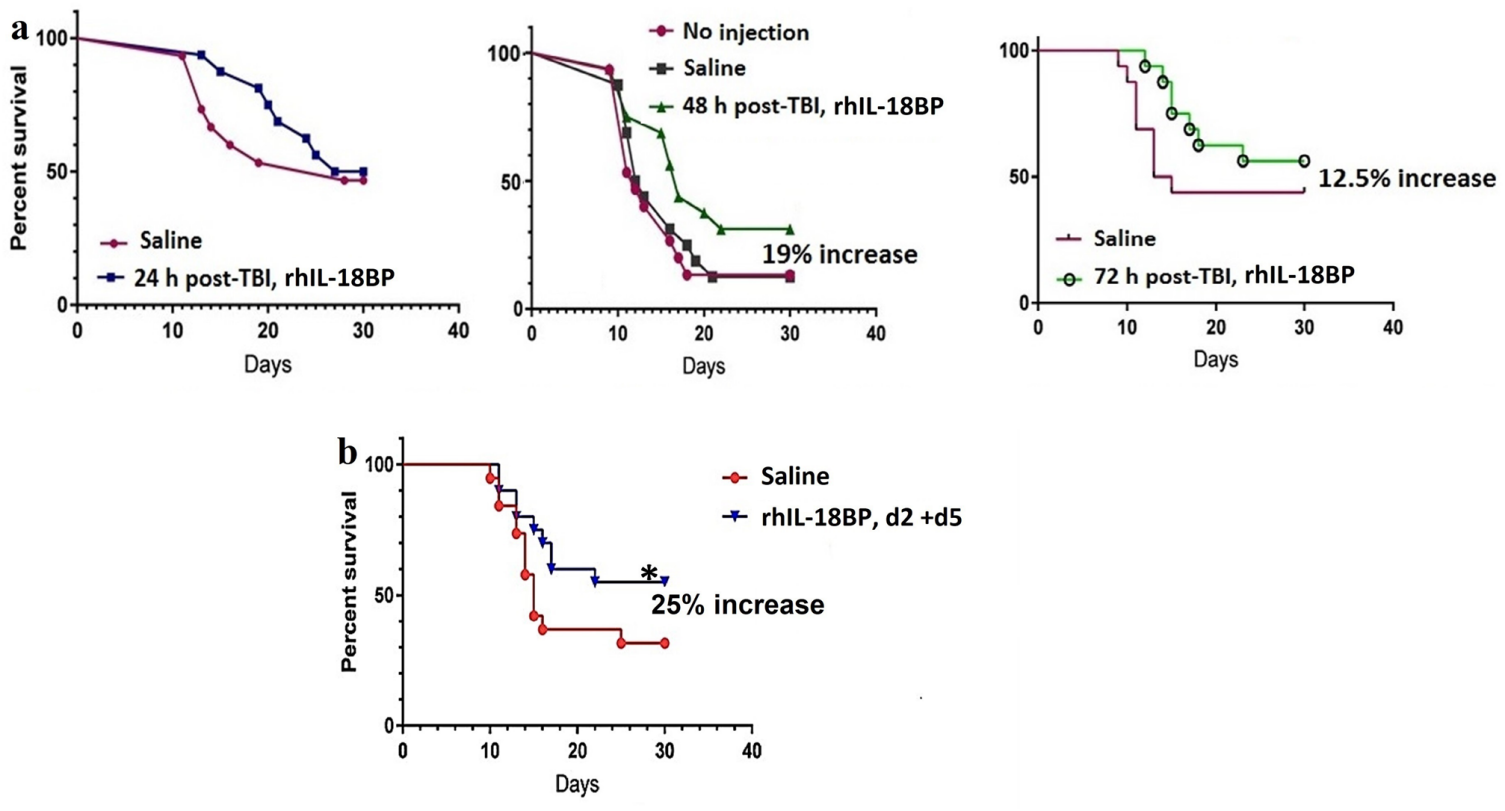
IL-18BP administration increased survival of mice after 9 Gy total-body irradiation (TBI). Recombinant human IL-18BP (1.5 mg/kg) or saline as a vehicle control was administered as a single (**a**) subcutaneous (SC) injection to mice 24, 48 or 72 h after 9 Gy TBI or double (**b**) SC injections at 2 and 5 days after 9 Gy TBI at a dose rate of 0.6 Gy/min (N = 20/group). Thirty-day survival was monitored after gamma radiation exposure. Survival rate for IL-18BP-treated groups were compared to vehicle-treated control groups.

Since no toxicity effects were observed from our toxicity study, we decided to use 1.5 mg/kg of rhIL-18BP for 30-day survival study according to the IL-18BP dose used in human clinical studies^24^. Single dose rhIL-18BP (1.5 mg/kg) or saline as vehicle control was administrated SC 24, 48 or 72 h after 9 Gy of TBI in CD2F1 mice. The 30-day mortality in vehicle- and rhIL-18BP-treated mice was calculated using Kaplan–Meier analysis. Results in Fig. 1a showed that giving a single injection of rhIL-18BP to mice at 24 h post-TBI exhibited a delayed mortality time in comparison with the vehicle control-treated mice. Administration of rhIL-18BP 48 h or 72 h post-TBI increased the survival of irradiated mice by 19.0% and 12.5%, respectively.

Our data above suggested that to increase survival rate in total-body irradiated mice, a multiple-dose administration of IL-18BP might be necessary. Next, rhIL-18BP (1.5 mg/kg) was given on day 2 and day 5 (two doses) after 9 Gy TBI. Choosing 2 days (48 h) after TBI as the first injection time was based on our data from the initial single injection experiment (Fig. 1a), where rhIL-18BP administration at 48 h post-TBI resulted in the highest survival rate increase (19% higher than control), and the second injection was given three days after the first injection. Results from the 30-day survival study shown in Fig. 1b demonstrated a significant survival rate (25% higher than control; p < 0.05) increase in mice administered two doses of IL-18BP compared with the vehicle control mice.

### IL-18BP increased blood platelets and BM hematopoietic progenitor cells in 30-day surviving mice after TBI

**Figure 2:**
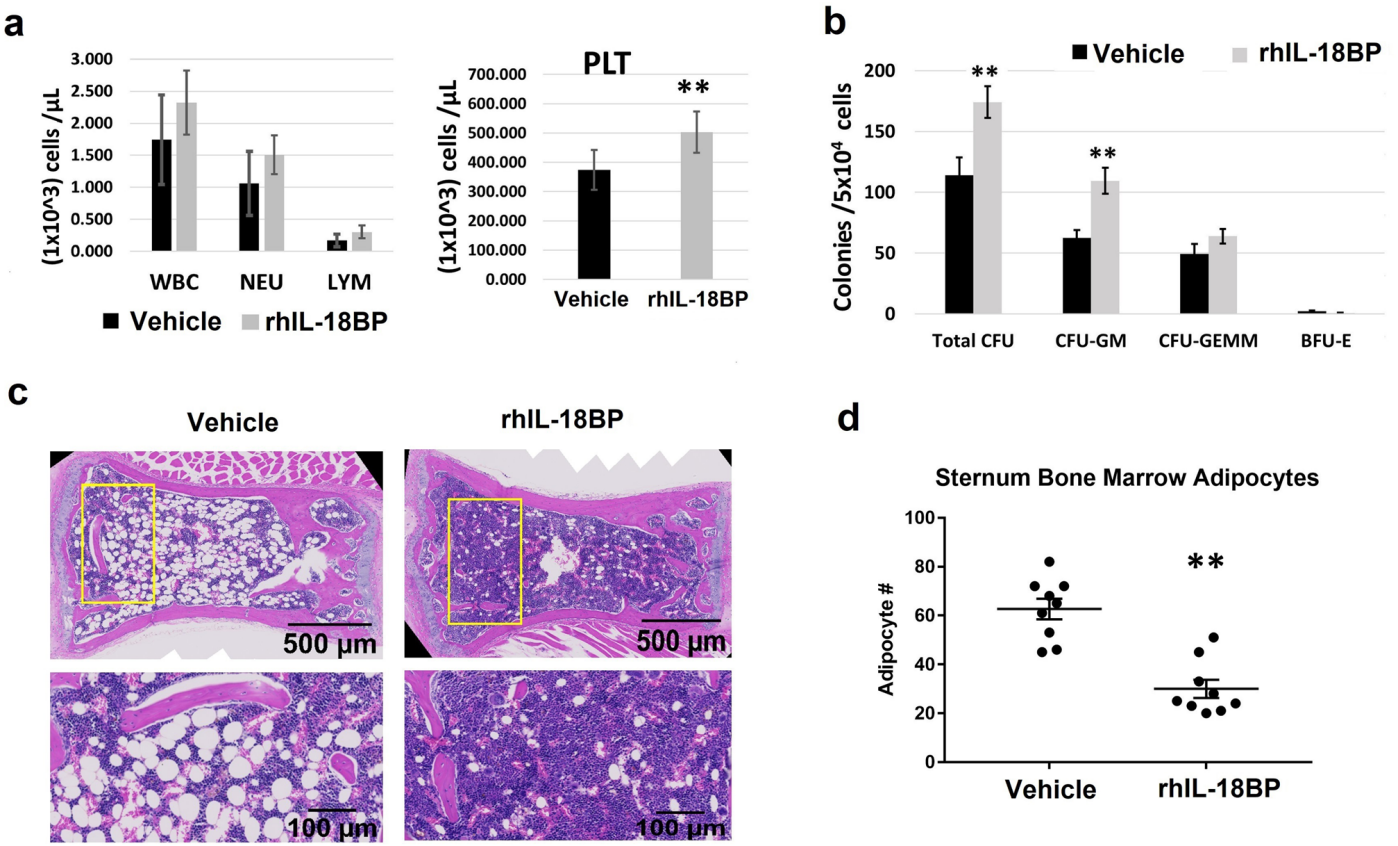
IL-18BP increased blood platelets and BM hematopoietic progenitor cells in 30-day survived mice after TBI. Mice received rhIL-18BP (1.5 mg/kg) or vehicle-injection at 48 h and 5 days after 9 Gy TBI. (**a**) Total white blood cells (WBC), neutrophil (NEU), lymphocyte (LYM), and platelets (PLT) were measured in whole blood samples from 30-day survived mice treated with rhIL-18BP or vehicle-injection after TBI. (**b**) Clonogenicity of mouse BM cells from these 30-day survived mice was also quantified in standard semisolid cultures in triplicate. CFU-GM, BFU-E and CFU-GEMM were counted 10 days later (6 mice/group). Means ± SD. Level of increase **p < 0.01; IL-18BP-treated vs. vehicle-treated. (**c**) HE staining of bone marrow from rhIL-18BP-treated and vehicle control-treated mice 30 days post-irradiation: longitudinal sections of entire sterna from representative mice in different groups at 20X and 40X magnification are shown; (**d**) Quantification of adipocytes in sternum BM sample treated with vehicle and IL-18BP 30 days post TBI are shown. Data represent mean ± SD. **p < 0.01.

Blood cell counts and BM clonogenicity from surviving mice 30 days after 9 Gy radiation exposure were evaluated (N = 6/group). Figure 2a showed the total white blood cells, neutrophils, lymphocytes and platelets measured in whole blood of mice that received rhIL-18BP or vehicle-injection at 48 h and 5 days post-TBI. The number of platelets was significantly increased from 374 ± 68 × 10^3^ cells/μL (saline control injected) to 503 ± 71 × 10^3^ cells/μL (rhIL-18BP injected) 30 days after irradiation. No difference of white blood cell, neutrophil, lymphocyte numbers was observed in vehicle or rhIL-18BP-treated 30-day surviving animals.

We further determined the effects of IL-18BP on survival of these mice BM hematopoietic progenitor cells. BM cells were collected from femurs and humeri of 30-day surviving mice treated either with two doses of rhIL-18BP or vehicle control. Total live BM myeloid cells in the pooled samples from each mouse were measured with Trypan blue staining. Colony assay was performed with 5 × 10^4^ BM cells/each animal and clonogenicity was scored and compared among samples collected from vehicle-treated and rhIL-18BP-treated mice (N = 6/group) as shown in Fig. 2b. Two-dose rhIL-18BP (injected 2 and 5 days after 9 Gy TBI) significantly increased total-colony number in 30-day surviving animals with high CFU-GM colonies. The early stage progenitors CFU-GEMM were also increased by IL-18BP although it was not significant.

Histologic study was also performed. Pathological changes in BM from sectioned sternal samples from rhIL-18BP-treated and vehicle control-treated animals on day-30 post-irradiation were evaluated. Marrow cellularity was measured in microscopic fields for each sectioned sternum (3 animals/group). Consistent with clonogenicity data, rhIL-18BP-treatment dramatically increased marrow hematopoietic cell population compared with vehicle-treated animal samples in 30-day surviving mouse after 9 Gy lethal irradiation. Representative BM images in Fig. 2c showed that, surviving TBI mice in vehicle-treated group had a severe hypocellularity of hematopoietic cells in which the marrow space was largely bereft of hematopoietic cells and mainly consisted of adipocytes. Treatments with rhIL-18BP improved BM cellularity in TBI mice. Furthermore, the numbers of adipocyte in the selected rectangle area of BM images were counted, since an increase in adipocyte number in BM is secondary to a decrease in hematopoietic cells^39^. rhIL-18BP treated mice had markedly decreased adipocyte counts when compared to animals in vehicle control treated as shown in Fig. 2d (p < 0.01).

### Treatment with IL-18BP decreased free IL-18 levels in mouse serum and increased blood cell counts and BM clonogenicity 4 and 7 days after TBI

**Figure 3:**
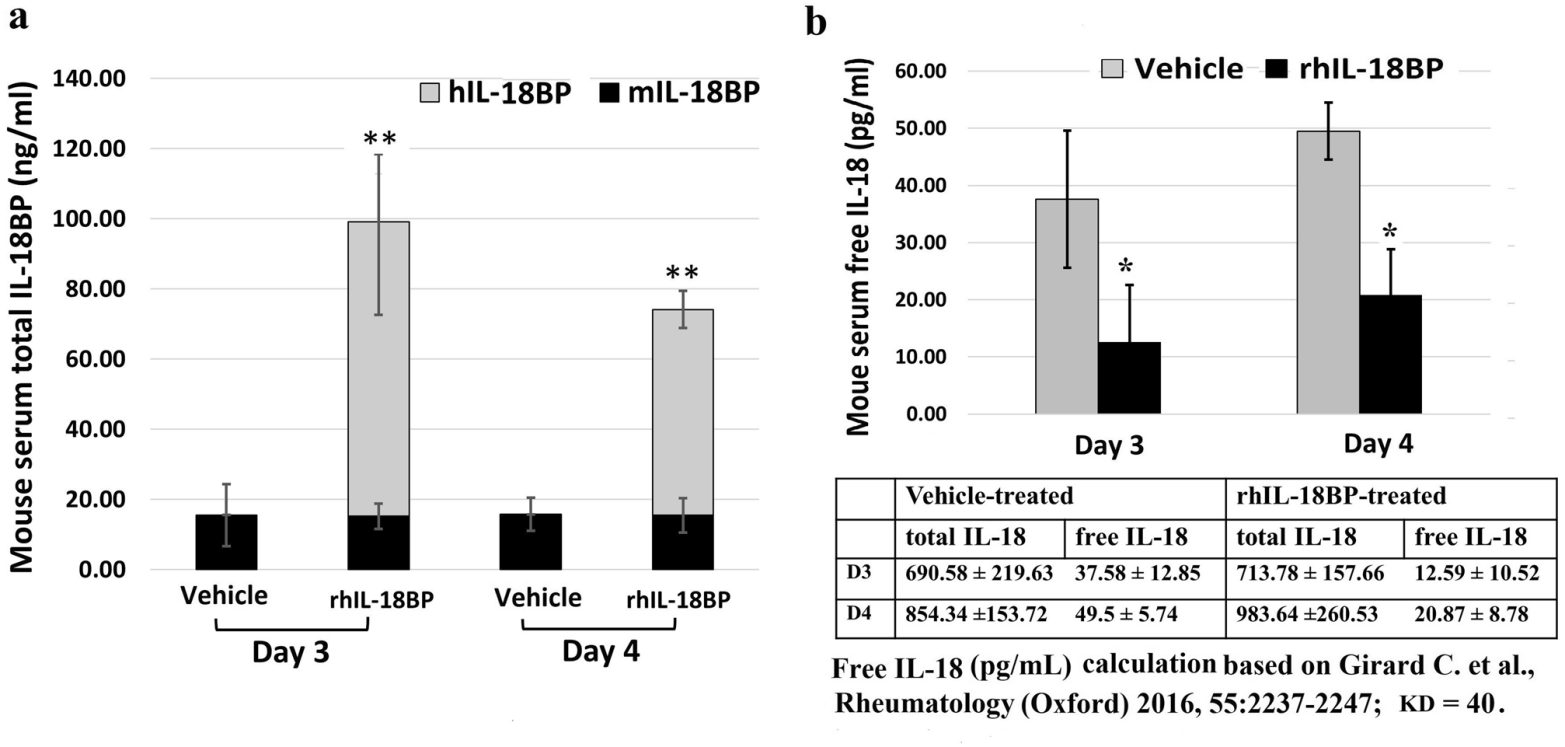
Treatment with IL-18BP decreased free IL-18 levels in mouse serum. Mice received single dose of rhIL-18BP (2 mg/kg) or vehicle-control SC injection 48 h post-9.4 Gy TBI and serum were collected on day 3 and 4 after TBI. Levels of mouse IL-18, endogenous mouse IL-18BP and exogenous human IL-18BP in mouse serum were measured with corresponding ELISA kits. (**a**) The total levels of IL-18BP (endogenous mouse IL-18BP and exogenous human IL-18BP) in vehicle-treated or rhIL-18BP-treated mouse serum and (**b**) free IL-18 in mouse serum. Data of total IL-18 in serum and free IL-18 levels by calculation in vehicle treated and IL-18BP treated group 3 and 4 day after TBI are included. Results were obtained from two independent experiments (6 mice/group/experiment, N = 12). Means ± SD. *p < 0.05; **p < 0.01; IL-18BP-treated vs. vehicle-treated.

Separate experiments were performed to test our hypotheses that administrating of rhIL-18BP could maintain balance of IL-18 and IL-18BP and decrease free IL-18 in mouse serum. Mice received single dose of rhIL-18BP (2.0 mg/kg) or vehicle-control SC injection 48 h post-9.4 Gy TBI. Mouse blood, serum and BM were collected 3, 4, and 7 days after TBI. Levels of mouse IL-18, endogenous mouse IL-18BP and exogenous human IL-18BP in mouse serum were measured by ELISA and free IL-18 in mouse serum were calculated. Data in Fig. 3a shows that SC injection of rhIL-18BP significantly increased mouse serum total IL-18BP (mouse + human IL-18BP) levels 3 and 4 days after TBI, and the free IL-18 levels calculated by Girard’s method in these mouse serum were significantly decreased compared with vehicle-control treated serum samples as shown in Fig. 3b.

**Figure 4:**
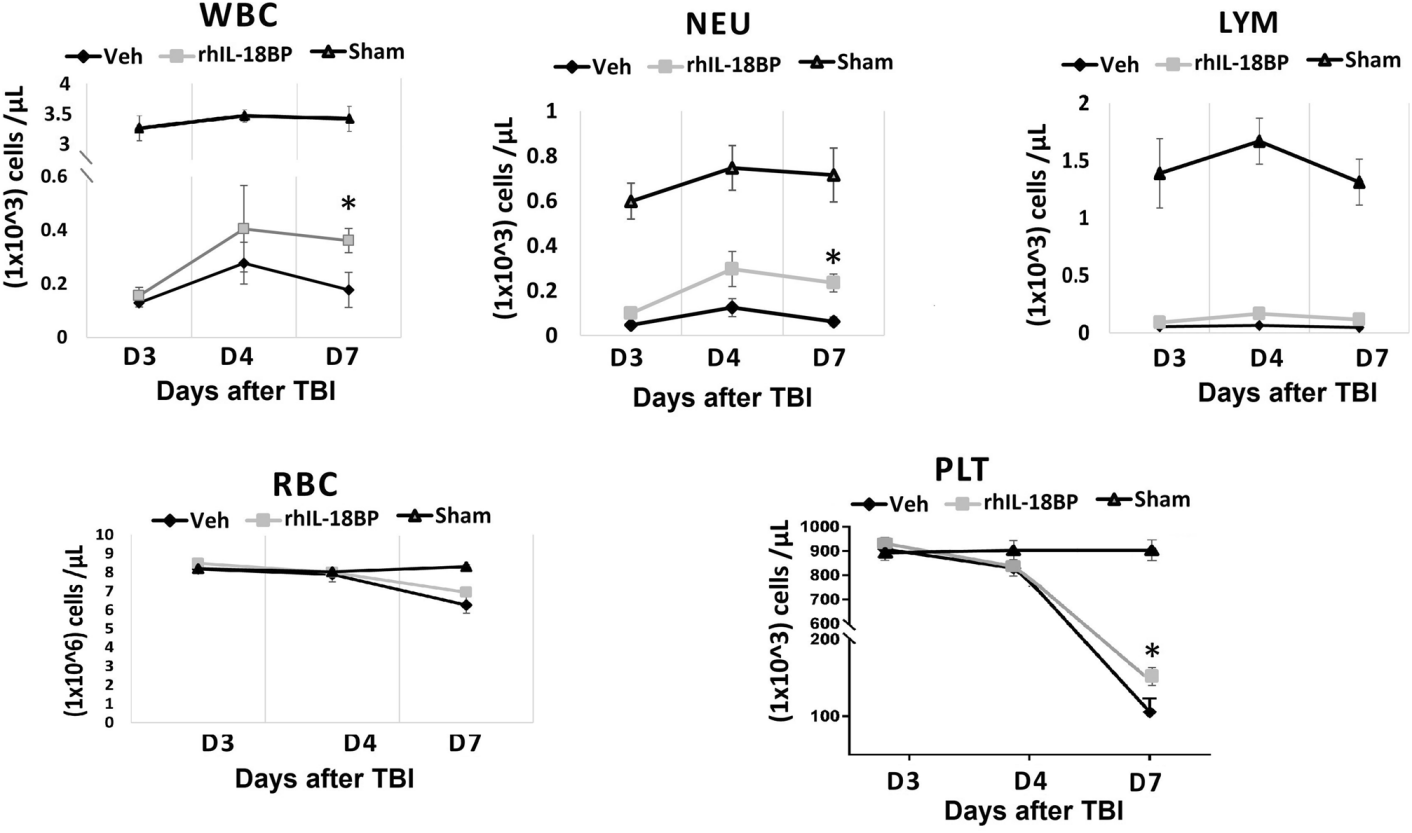
Treatment with rhIL-18BP increased blood cell counts 4 and 7 days after TBI. Mice received rhIL-18BP (2 mg/kg) or vehicle-injection at 48 h after 9.4 Gy TBI. Total white blood cells (WBC), neutrophil (NEU), lymphocyte (LYM), platelets (PLT) and red blood cells (RBC) were measured in whole blood samples 3, 4 and 7 days after TBI. Results were from two independent experiments (6 mice/group/experiment, N = 12). Means ± SD. Level of increase *p < 0.05; **p < 0.01; rhIL-18BP-treated vs. vehicle-treated.

**Figure 5:**
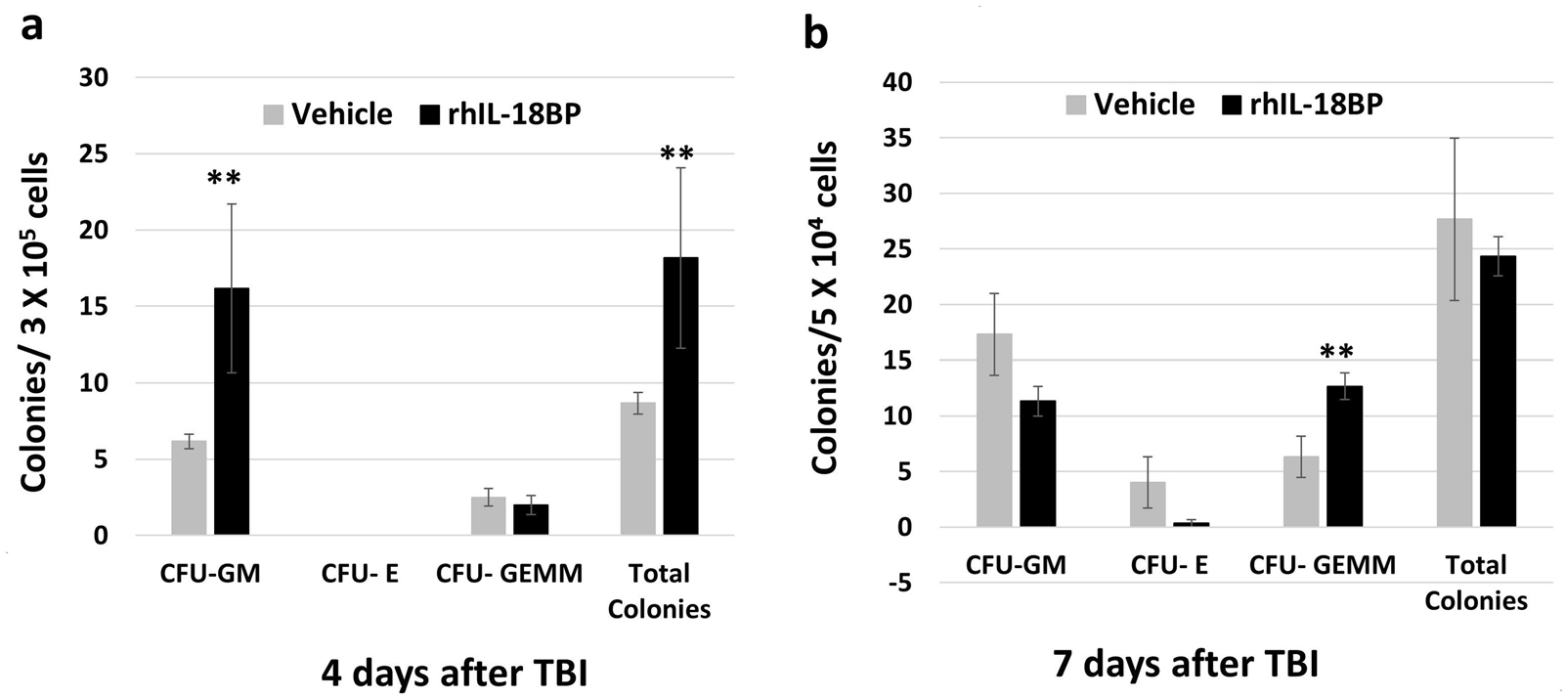
Treatment with IL-18BP increased BM clonogenicity 4 and 7 days after TBI. Mice received rhIL-18BP (2 mg/kg) or vehicle-injection at 48 h after 9.4 Gy TBI. Clonogenicity of mouse BM cells from 4 (**a**) and 7 (**b**) days after TBI was quantified in standard semisolid cultures in triplicates. CFU-GM, BFU-E and CFU-GEMM were counted 10 days later. Results were from two independent experiments (6 mice/group/experiment, N = 12). Means ± SD. Level of increase *p < 0.05; **p < 0.01; rhIL-18BP-treated vs. vehicle-treated.

We further measured blood cell numbers and BM clonogenicity in these mice. rhIL-18BP treatment significantly increased blood cell counts 7 days after irradiation (Fig. 4) including total white blood cells, neutrophils, and platelets; the lymphocytes and red blood cell number were not significantly changed by rhIL-18BP treatment in comparison with vehicle-control treated mice. Furthermore, rhIL-18BP treatment significantly increased total colony number with the most increase in CFU-GM increased on day-4 (Fig. 5a). On day 7 all colonies both in vehicle control and rhIL-18BP treated animals were higher than on day 4, and among them the early stage colonies CFU-GEMMs were significantly higher in rhIL-18BP-treated than vehicle-treated mice as shown in Fig. 5b.

### Treatment with IL-18BP inhibited the IL-18 downstream target IFN-γ expression in mouse BM after TBI

**Figure 6:**
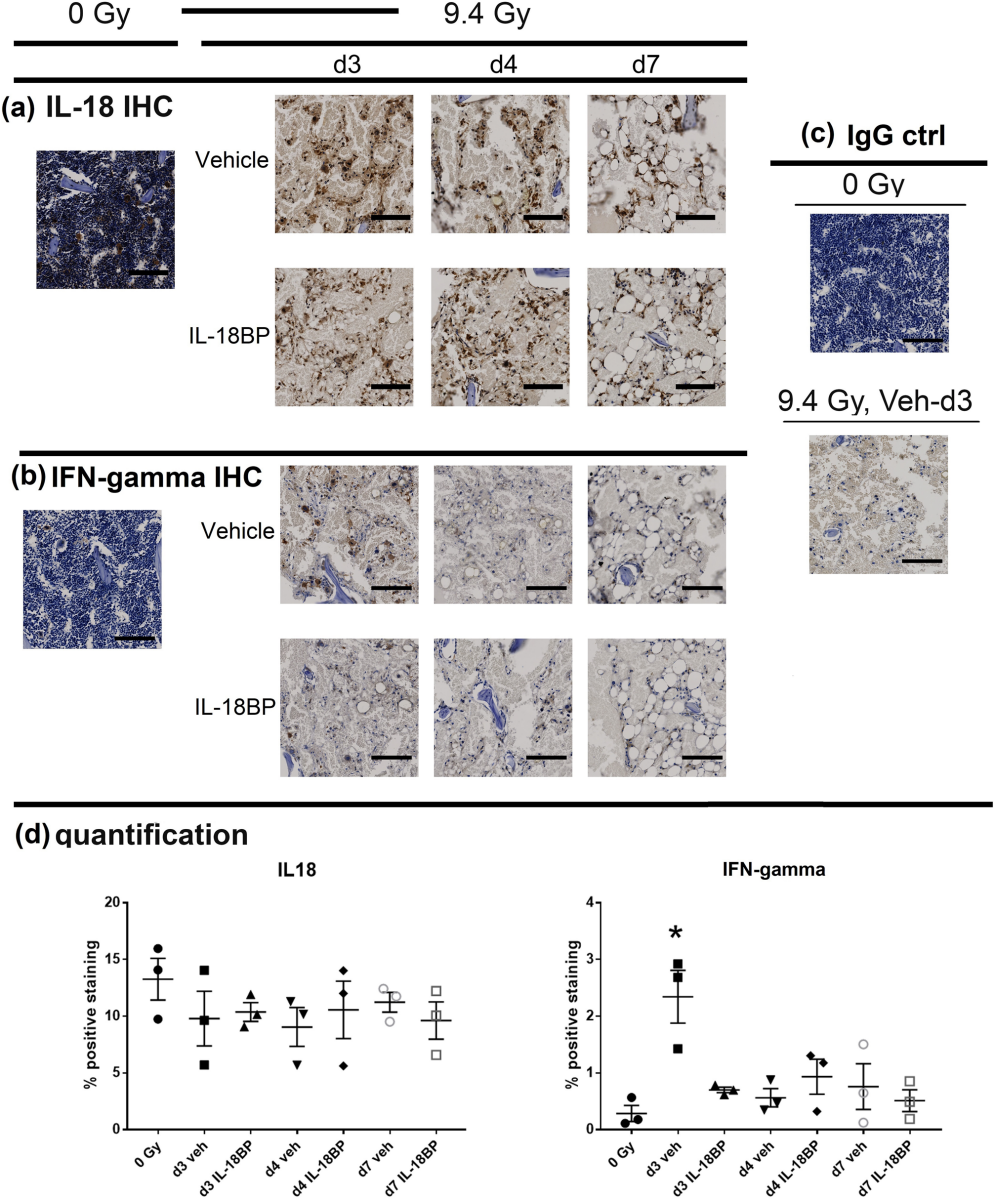
Immunohistochemical (IHC) staining of bone marrow with anti-IL-18 and IFN-γ antibodies. Mice were irradiated with 9.4 Gy TBI and then treated with vehicle or IL-18BP at 48 h post TBI. Mice were sacrificed at day (d) 3, d4 and d7 after TBI. Un-irradiated mice were included for comparison. Sternum bone marrow specimens were stained with IL-18 (**a**), IFN-γ (**b**) or rabbit IgG (**c**) antibodies. Brown color indicates positive staining. Scale bar = 100 µm. Quantification of IL-18 and IFN-γ IHC staining was shown in (**d**), N = 3.*p < 0.05. Note that the d3 vehicle treated group was significantly different with any other group in the IFN-γ staining.

Mice received a single dose of rhIL-18BP (2.0 mg/kg) or vehicle-control SC injection 48 h post-9.4 Gy TBI. Immunohistochemistry (IHC) staining with antibodies to identify IL-12, IL-15, IL-18, IFN-γ and TNF-α was conducted in mouse sectioned sternal BM samples from vehicle control-treated and rhIL-18BP-treated animals as described in Material and Methods. Samples from sham (0 Gy)-irradiated mouse BM were be used as control. As shown in Fig. 6, brown color represents positive DAB staining and sections stained with rabbit IgG isotype control antibody has no DAB staining(Fig. 6c). Figure 6a shows anti-IL-18 antibody stained samples: in sham-irradiated BM sample, there were cells constitutively expressing IL-18. Most of these cells were megakaryocytes, but there were also small size BM cells. After 9.4 Gy TBI, most BM hematopoietic cells were depleted, and the levels of IL-18 positive staining in vehicle-treated samples were high in different type of BM stroma and niche cells 3 and 4 days post-TBI. On day 7 after TBI, IL-18 expression was decreased and there were more adipocytes in the BM than earlier time points. IL-18BP treatment had no significant effect on IL-18 staining (Fig. 6d), since the anti-IL-18 antibody used in this assay cannot distinguish the free-IL-18 from IL-18BP bound IL-18.

For anti-IFN-γ antibody staining as shown in Fig. 6b, a few IFN-γ positive cells were observed in 0 Gy-irradiated BM cells. However, there was significantly increased IFN-γ positive staining in vehicle treated BM on day 3 and went back to baseline level on day 4 and 7 after 9.4 Gy TBI. Treated with IL-18BP, the IFN-γ positive staining was significantly less on day 3 after 9.4 Gy TBI compared to the vehicle group (Fig. 6d). IL-18BP- treated groups on day 4 and 7 had similar levels of IFN-γ positive staining with the vehicle groups. There were very low level of IL-15, IL-12 and TNF-α staining under all conditions (data not shown).

### Treatment with IL-18BP decreased reactive oxygen species (ROS) levels in the mouse heart tissues after TBI

**Figure 7:**
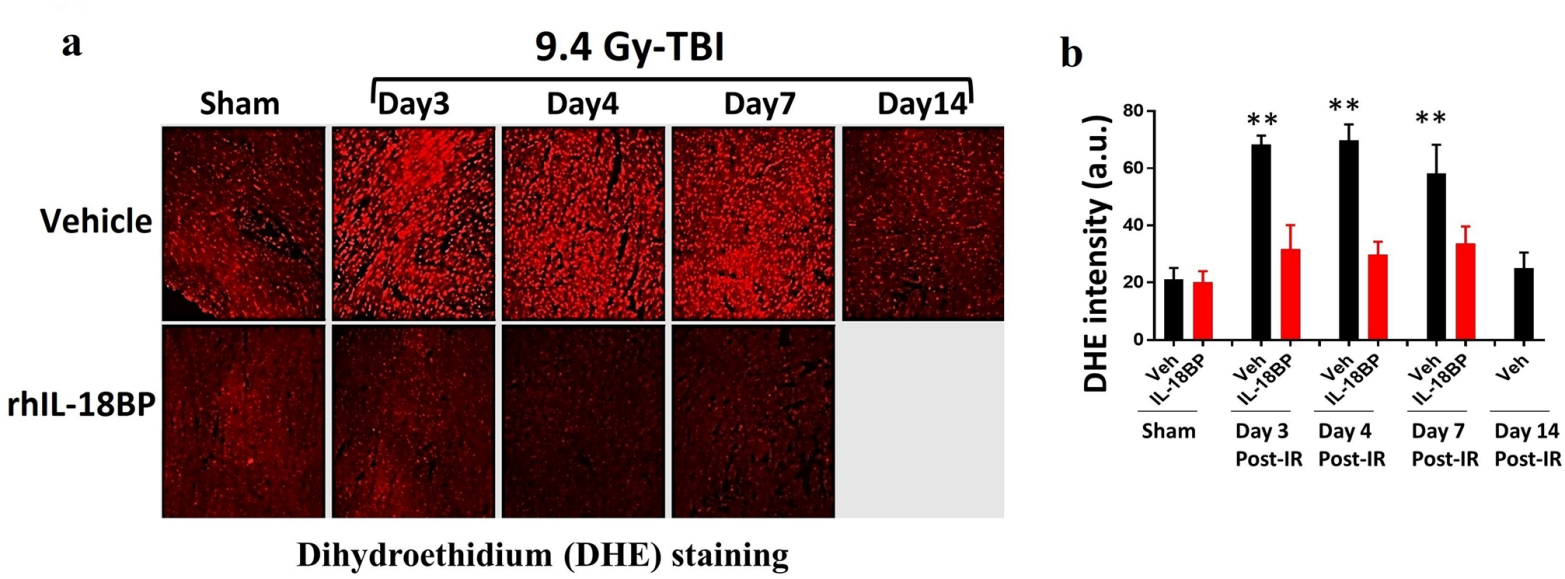
Treatment with IL-18BP inhibited ROS levels in irradiated mouse heart tissue. Mice received single dose of rhIL-18BP (2 mg/kg) or vehicle-control SC injection 48 h post- 9.4 Gy TBI and heart tissues were collected on day 3, 4, 7 and 14 after TBI. (**a**) Representative fluorescent microscopy images of fresh frozen section of mouse heart labeled with DHE. (**b**) DHE fluorescence intensity was quantified as the ROS levels. 40 cardiomyocytes were randomly selected from 6–8 images in each group (N = 6 mice/group). Means ± SD. Level of increase **p < 0.01; rhIL-18BP-treated vs. vehicle-treated.

Mice received a single dose of rhIL-18BP (2.0 mg/kg) or vehicle-control SC injection 48 h post-9.4 Gy TBI. Mouse heart tissues were collected on day 3, 4, 7, and 14 after TBI and ROS levels were determined. ROS are short-lived molecules, which makes them difficult to be directly measured in vivo. Thus, we and other researchers have used dihydroethidium (DHE) staining to visualize ROS levels in freshly frozen tissue sections^35,40^. Figure 7a shows that the DHE fluorescence intensities were significantly elevated in mouse heart tissues collected on day 3, 4 and 7 after TBI, and returned to similar level as sham-irradiated on day-14. More importantly, rhIL-18BP treatment greatly dampened ROS increase associated with irradiation on day 3, 4 and 7. The fluorescence intensity was summarized in Fig. 7b. rhIL-18BP treatment significantly decreased the DHE fluorescence intensity levels at all time points after irradiation, which suggests that IL-18BP treatment may ameliorate radiation-induced tissue injuries potentially through reducing ROS production.

### IL-18BP decreased growth differentiation factor-15 (GDF15) and increased citrulline levels in mouse serum after TBI

**Figure 8:**
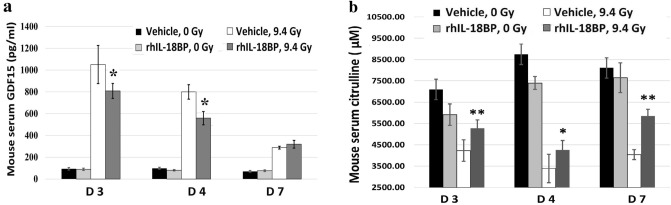
IL-18BP decreased GDF15 and increased citrulline levels in mouse serum after TBI. Mice received single dose of rhIL-18BP (2 mg/kg) or vehicle-control SC injection 48 h post-sham (0 Gy) or 9.4 Gy TBI and sera were collected on day 3, 4 and 7 after TBI. Levels of (**a**) GDF15 and (**b**) citrulline in mouse serum were measured with ELISA and Colorimetric kit. Results were obtained from two independent experiments (6 mice/group/experiment, N = 12). Means ± SD. Level of increase *p < 0.05; **p < 0.01; rhIL-18BP-treated vs. vehicle-treated.

The effects of rhIL-18BP on regulation of the inflammatory and stress responsive factor GDF-15 and intestinal injury biomarker citrulline levels in irradiated mouse serum (N = 6 mice /group) were evaluated. Vehicle control or rhIL-18BP (2 mg/kg) was injected 48 h after 0 or 9.4 Gy TBI and mouse serum were collected on day 3, 4 and 7 after TBI. Levels of GDF15 in the serum of individual mice were measured by ELISA as described in “Materials and methods”. In sham irradiated mice, rhIL-18BP treatment did not affect the serum GDF-15 levels and the average GDF-15 level was 76.4 ± 19.67 pg/mL. Radiation significantly increased the GDF15 levels in mouse serum up to 1,029.7 ± 110.6 pg/mL and 800 ± 100.7 pg/mL 3 and 4 days after TBI in vehicle-treated groups, respectively; by day 7, the level of GDF-15 in irradiated mouse serum dropped to 267.47 ± 26.27 pg/mL. Administration of rhIL-18BP significantly decreased the GDF-15 levels in mouse serum to 792.12 ± 106.15 pg/mL on day 3 and 561.82 ± 54 pg/mL on day 4 post-TBI as shown in Fig. 8a.

Next, we evaluated the noncoded amino acid citrulline levels in these mouse serum using colorimetric kit since citrulline is produced almost exclusively by the enterocytes of the small intestine and has been identified as a simple, sensitive and suitable biomarker for radiation-induced gastrointestinal-acute radiation syndrome (GI-ARS)^41^. Data in Fig. 8b indicated that levels of citrulline in sham irradiated and vehicle or rhIL-18BP treated animal serum were 7,983 ± 371 µM. No significant differences of citrulline levels were observed in IL-18BP treated and vehicle treated animals after sham irradiation. Radiation at 9.4 Gy decreased the mouse citrulline levels to 3,884 ± 469 µM in vehicle control treated group, whereas treatment with rhIL-18BP maintained the serum citrulline levels at 5,130 ± 380 µM after 3–7 days of TBI. The difference of citrulline levels between vehicle-treated and rhIL-18BP-treated mice was significant at all time points after TBI (Fig. 8b).

## Discussion

Currently, three white blood cell growth factors (Neupogen, Neulasta and Leukine) have been approved by the FDA for mitigating hematopoietic acute radiation syndrome (H-ARS)^42^. However, these growth factors are associated with side effects such as bone pain^43^ and their mitigative efficacy only observed by giving the drug within 24 h post-irradiation in irradiated mice and nonhuman primates^44,45^. We aimed to develop new therapies for ARS with high efficacy, no side effects and wider therapeutic window since the chance of receiving treatment after radiation injury varies with availability of the medical support, most likely beyond 24 h post-radiation exposure. Recent studies suggested that radiation exposure causes local and systemic inflammatory responses and induces cell and tissue damage^46^. IL-18 is an IL-1 family member produced by various hematopoietic and nonhematopoietic cells and plays key roles in inflammatory and immune responses^8,9^; and IL-18BP, a natural antagonist of IL-18, has been used safely and effectively in clinical trials and animal models to treat IL-18-related inflammatory diseases characterized by severe, life threatening systemic inflammation associated with extremely high levels of IL-18^23,24^. Recently, Belkaya et al.^14^ reported a child died from infection of hepatitis A virus (HAV) due to homozygous *IL-18BP* gene mutation and absence of IL-18BP in her body caused uncontrolled high level IL-18-induced excessive NK cell activation and subsequently killed both HAV infected and healthy hepatocytes. This report further indicated that keeping IL-18/IL-18BP balance plays an important role in healthy individuals.

In the current study, we evaluated the levels of inflammatory factor IL-18 and its natural antagonist IL-18BP in mouse serum 1 and 3 days after 0.5–10 Gy total-body ^60^Co gamma irradiation (TBI) and demonstrated that radiation-induced IL-18 in mouse serum was significantly increased in a radiation dose-dependent manner. In addition, our data further suggested an exhausted IL-18BP level in mouse serum after > 3 Gy TBI which resulted in an imbalance of IL-18 to IL-18BP and a dramatic increase of free IL-18 in mouse serum 3 days after TBI as shown in Table 1. Based on our data, we hypothesized that administration of IL-18BP after TBI could boost the endogenous IL-18BP levels in animal serum and mitigate radiation-induced cell and tissue damage and subsequently increase survival of mice after lethally radiation exposure. Results in Fig. 3a demonstrate that SC injection of rhIL-18BP 48 h post-TBI significantly increased total IL-18BP levels and suppressed the free IL-18 in mouse serum on day 3 and day 4 post-irradiation. We further tested the mitigative efficacy of rhIL-18BP on survival of mice after lethal cobalt radiation exposure. Thirty-day survival results in Fig. 1 showed that giving single injection of rhIL-18BP to mice at 24 h after 9 Gy TBI exhibited a delayed mortality time in comparison with vehicle control-treated mice; administrating of rhIL-18BP 48 h or 72 h post-TBI increased the 30-day survival of irradiated mice by 19% and 12.5%, respectively. Furthermore, a 25% survival rate increase compared with vehicle control group was observed in mice received rhIL-18BP-treatment on day 2 and day 5 after 9 Gy TBI. Our data suggested that to increase survival rate in total-body irradiated mice, a multiple-dose administration of rhIL-18BP might be necessary although the optimal treatment dose/schedule of rhIL-18BP is under investigation in our lab.

Treating the radiation exposed mice with rhIL-18BP significantly mitigated their blood and BM injury, resulted in increased blood cell counts and progenitor cell colonies at early time (3–7 days) and 30 days post-TBI (Figs. 2, 4 and 5). In addition, hematopoietic cellularity increased by IL-18BP treatment was also observed in 30-day survived animals’ BM histologic samples (Fig. 2c). Our data indicated that IL-18BP treatment could mitigate the radiation-induced acute hematopoietic syndrome. Radiation-induced hematopoietic cell damage and death through apoptosis and non-apoptotic modes of programmed cell death such as necroptosis and pyroptosis which are involved in the inflammatory pathway and the insufficiency of BM recovery after radiation exposure has been directly correlated with subsequent hematopoietic and immune system depletion. IL-18 and its downstream pre-inflammatory factors play an important role in these pathways. To understand the mechanism by which IL-18BP plays mitigative function in irradiated mouse BM, we conducted IHC staining with anti-IFN-γ, anti-IL-12, anti-IL-15, anti-IL-18 and anti-TNF-α antibodies on sternum sections from mice after 9.4 Gy TBI and treated with vehicle control or 2 mg/kg IL-18BP 48 h post-TBI. Histologic image in Fig. 6 demonstrated that total TBI caused severe BM hypocellularity of hematopoietic cells and persisted IL-18 expression post-TBI compared with sham irradiated samples. There was no significant difference of IL-18 staining between vehicle group and IL-18BP treatment group after radiation, because although IL-18 was sequestered by IL-18BP, the anti-IL-18 antibody still could recognize it in that location and not degraded. Most importantly, IL-18BP treatment significantly inhibited the IL-18 downstream target IFN- γ expression in mouse BM 3 days after TBI. IL‐18 was originally discovered as an IFN-γ-inducer which induces IFN-γ production from Th1 and NK cells with or without IL-12 upon stimulation with antigens^47,48^. IFN-γ is an important inflammatory factor which induced inflammatory response and ROS can cause cell death through apoptosis and necroptosis^49,50^. We tested the expression of potential synergistic mediators of IL-18 signaling, such as IL-15 and IL-12, in irradiated BM. Our data did not suggest IL-15 and IL-12 were important here. TNF-α, which is a potential downstream factor underneath IL-18, was not elevated in irradiated BM or changed with IL-18BP treatment. Our data suggested that the radiation mitigative effect of IL-18BP on survival of mouse BM hematopoietic cells may act through inhibiting IL-18 and its downstream inflammatory factor IFN-γ.

Accumulated evidences indicated that clinical radiotherapy in patients’ chest can affect the heart, blood vessels and lungs, and radiation-induced heart disease (RIHD) is a major source of morbidity and mortality^51,52^. Radiation-induced pro-inflammatory cytokines such as IFN-γ can increase ROS production via mitochondria and NADPH oxidase^53^. ROS are important signaling molecules that play a key role in inflammation-mediated tissue injuries^54^. To explore the underlying mechanisms of mitigative effects of IL-18BP after irradiation, we evaluated ROS levels in mouse heart tissues at different time points after TBI with and without IL-18BP treatment. Our data in Fig. 7 showed that the ROS levels were significantly elevated in mouse hearts after radiation exposure and IL-18BP treatment greatly suppressed this radiation-induced ROS, presumably through inhibiting the IL-18-IFN- γ -ROS pathway. ROS are short-lived molecules, which makes them difficult to be directly measured in vivo. In this study, we used DHE staining to visualize ROS levels in freshly frozen heart tissue sections based on our and others’ reports^35,40^. We acknowledge this limitation when interpreting our findings. Further studies are needed to confirm the ROS results and assess oxidative stress, i.e., 4-Hydroxynonenal for lipid oxidation or 8-oxoguanine for DNA oxidative damage.

Furthermore, GDF-15 is a stress responsive cytokine belonging to the TGF-beta/bone morphogenetic protein (BMP) superfamily and highly expressed in cardiomyocytes, adipocytes, macrophages, endothelial cells, and vascular smooth muscle cells. GDF-15 increases during tissue injury and inflammatory states and is associated with cardiometabolic risk^55,56^. Together, data from our current study demonstrated that lethal doses of TBI caused increases of ROS in mouse heart tissue and GDF-15 in mouse serum, suggesting the radiation-induced heart and vascular tissue injuries have happened to these animals. Interestingly, injection of rhIL-18BP 48 h post-radiation could significantly decrease the radiation-induced ROS and GDF-15 levels after TBI, implicating that IL-18BP may protect heart tissue from radiation injuries.

In addition, data from our study also demonstrated that administration of rhIL-18BP could help mice recover from TBI-caused low citrulline levels in their serum. The noncoded amino acid citrulline is synthesized almost exclusively by the enterocytes of the small intestine and plasma citrulline has been identified as a biomarker of the functional small bowel enterocytes^57^. Radiation induced damage to the gastrointestinal tract is characterized by intestinal crypt cell apoptosis and mitotic cell death leading to loss of mucosal barrier function, villous atrophy, alterations in the normal intestinal bacterial flora and enterocyte mass reduction^28^. Citrullinemia has been identified as a simple, sensitive and suitable biomarker for radiation-induced injury associated with gastrointestinal-acute radiation syndrome (GI-ARS)^41^. Our data demonstrated that IL-18BP protected radiation-induced reduction of citrulline in mouse serum.

In conclusion, our data suggest that IL-18 plays a key role in radiation-induced cell and tissue damage and dysfunction through IL-18/IFN-γ and/or ROS inflammatory pathway. IL-18BP counters IL-18 activation and therefore may mitigate/treat radiation-induced multiple organ injuries and increase survival with a wider therapeutic window from 24 h and beyond after lethal doses of radiation exposure. The optimal therapeutic dose of IL-18BP for radiation injury in experimental mice and the effects of IL-18BP on radiation-induced mouse gastrointestinal and heart injuries are under further investigation in our laboratory.

## Data Availability

All data generated or analyzed during this study are included in this published article.
